# Airborne Multidrug-Resistant Bacteria Isolated from a Concentrated Swine Feeding Operation

**DOI:** 10.1289/ehp.7473

**Published:** 2004-11-22

**Authors:** Amy Chapin, Ana Rule, Kristen Gibson, Timothy Buckley, Kellogg Schwab

**Affiliations:** Johns Hopkins Bloomberg School of Public Health, Department of Environmental Health Sciences, Baltimore, Maryland, USA

**Keywords:** air sampling, airborne bacteria, antibiotic resistance, CAFO, concentrated swine feeding operation, multidrug-resistant bacteria

## Abstract

The use of nontherapeutic levels of antibiotics in swine production can select for antibiotic resistance in commensal and pathogenic bacteria in swine. As a result, retail pork products, as well as surface and groundwaters contaminated with swine waste, have been shown to be sources of human exposure to antibiotic-resistant bacteria. However, it is unclear whether the air within swine operations also serves as a source of exposure to antibiotic-resistant bacterial pathogens. To investigate this issue, we sampled the air within a concentrated swine feeding operation with an all-glass impinger. Samples were analyzed using a method for the isolation of *Enterococcus*. A total of 137 presumptive *Enterococcus* isolates were identified to species level using standard biochemical tests and analyzed for resistance to erythromycin, clindamycin, virginiamycin, tetracycline, and vancomycin using the agar dilution method. Thirty-four percent of the isolates were confirmed as *Enterococcus*, 32% were identified as coagulase-negative staphylococci, and 33% were identified as viridans group streptococci. Regardless of bacterial species, 98% of the isolates expressed high-level resistance to at least two antibiotics commonly used in swine production. None of the isolates were resistant to vancomycin, an antibiotic that has never been approved for use in livestock in the United States. In conclusion, high-level multidrug-resistant *Enterococcus*, coagulase-negative staphylococci, and viridans group streptococci were detected in the air of a concentrated swine feeding operation. These findings suggest that the inhalation of air from these facilities may serve as an exposure pathway for the transfer of multidrug-resistant bacterial pathogens from swine to humans.

The development and persistence of multidrug-resistant bacteria pose increasing challenges to public health (Institute of Medicine 1998). Although the use of antibiotics in human medicine has influenced the emergence of antibiotic-resistant bacteria, the use of antibiotics in animal agriculture has markedly contributed to this critical problem as well (Cohen and Tauxe 1986; Gorbach 2001; Institute of Medicine 1998; National Research Council 1999; van den Boogard and Stobberingh 1999). In animal agriculture, antibiotics are administered for therapeutic purposes to treat infections, prophylactic purposes in advance of observed symptoms, and nontherapeutic purposes to promote growth and improve feed efficiency (Wegener 2003). In general, antibiotics are administered at higher concentrations for therapeutic and prophylactic use and lower concentrations for nontherapeutic use (Wegener 2003). It has been estimated that the nontherapeutic use of antimicrobials in livestock production comprises 60–80% of total antimicrobial production in the United States (Mellon et al. 2001). The swine industry alone uses an estimated 10.3 million pounds of antibiotics annually for nontherapeutic purposes. Among the antibiotics used are ampicillin, bacitracin, erythromycin, lincomycin, virginiamycin, and tetracycline (Food and Drug Administration 2004), some of which are important in human clinical medicine. The use of antibiotics for nontherapeutic purposes such as growth promotion has been shown to select for resistance to high concentrations of antibiotics in both pathogenic and commensal bacteria in swine (Aarestrup et al. 2000a, 2000b; Bager et al. 1997; Jensen et al. 2002; Wegener et al. 1999). For this reason, attention has been given to retail pork products as a source of human exposure to antibiotic-resistant bacteria (Donabedian et al. 2003; Gambarotto et al. 2001; Hayes et al. 2003; Sorensen et al. 2001; White et al. 2001). Yet the ingestion of pork products is not the only pathway of exposure for the transfer of resistant organisms from swine to humans. Environmental pathways of exposure may be equally important.

Along with the pork products, more than 110 million tons of swine waste—containing antibiotic-resistant bacteria—is produced at swine concentrated animal feeding operations (CAFOs) in the United States each year (Environmental Defense 1997). The practice of storing this waste in pits and open-air lagoons and subsequently applying the waste to land can lead to the contamination of soils and nearby surface and groundwaters. Several studies have reported the appearance of antibiotic residues and antibiotic-resistant bacteria in surface and groundwaters proximal to swine CAFOs (Campagnolo et al. 2002; Chee-Sanford et al. 2001). Campagnolo et al. (2002) suggested that swine waste may be a source of antimicrobial drugs in surface and groundwaters near swine facilities, and Chee-Sanford et al. (2001) found that groundwater can be affected by swine waste and serve as a potential source of exposure to antibiotic-resistance genes.

However, few studies have examined the air within swine CAFOs as an additional source of environmental exposure to antibiotic-resistant bacterial pathogens. It has been well documented that the air within swine CAFOs is highly contaminated with bacteria, yeasts, and molds. Mean total bacterial concentrations can range from 10^4^ to 10^7^ colony forming units (CFU)/m^3^ (Clark et al. 1983; Cormier et al. 1990; Crook et al. 1991; Predicala et al. 2002). Specific bacteria detected in the air of swine CAFOs have included the following potential human pathogens: *Enterococcus*, *Staphylococcus*, *Pseudomonas*, *Bacillus*, *Listeria*, and *Escherichia coli* (Cormier et al. 1990; Crook et al. 1991; Predicala et al. 2002). Yet, to date, these airborne pathogens have not been assessed for resistance to antibiotics that are commonly used in both swine production and clinical medicine. Hamscher et al. (2003) assessed the presence of antibiotics in dust samples collected at a swine production facility over two decades. Several different antibiotics, including tetracycline, tylosin (an analog to erythromycin), and chloramphenicol, could be detected in 90% of the dust samples tested (Hamscher et al. 2003). In abstract form within conference proceedings, Zahn et al. (2001) reported on the presence of tylosin and tylosin-resistant bacteria in the air released from three mechanically ventilated swine CAFOs. Their study indicated that tylosin-resistant bacteria, primarily *Corynebacterium*, accounted for 80% of total culturable bacteria detected. These results provided the first evidence of airborne antibiotic-resistant bacteria in swine CAFOs.

The goal of this study was to test air samples collected within a swine CAFO for the presence of antibiotic-resistant enterococci, gram-positive, catalase-negative cocci that are not only members of the normal intestinal flora of humans and animals but also capable of causing a variety of human and animal infections [National Nosocomial Infections Surveillance (NNIS) 2001]. Resistance to erythromycin, clindamycin, tetracycline, and virginiamycin [an analog to quinupristin/dalfopristin, which is used to treat vancomycin-resistant *Enterococcus faecium* infections in humans (Johnson and Livermore 1999)] was investigated. These drugs (or their analogs) have been approved for use in swine production for growth promotion, feed efficiency, and therapeutic purposes. Resistance to vancomycin also was tested. Vancomycin, an analog to avoparcin, which has been used extensively in animal agriculture in Europe, has never been approved for use in livestock in the United States.

## Materials and Methods

### Study site.

The study site is a swine finishing CAFO located in the Mid-Atlantic United States. The CAFO consists of two tunnel-ventilated swine houses built atop 12-ft deep concrete pits where swine waste is stored before periodic siphoning into a transport truck for off-farm disposal by land application. Each house has the capacity to hold 2,500 hogs; however, during the sampling period approximately 1,500 hogs were being housed in each building. Air sampling at the swine facility was conducted on 9 December 2003 and 5 January 2004.

### Collection of air samples.

Air samples were collected at a calibrated flow rate of 12.5 L/min using all-glass impingers (AGI-30; Ace Glass, Vineland, NJ) designed to collect respirable particles, including bioaerosols, with an aerodynamic diameter < 5 μm. Impingers were autoclaved and filled with 20 mL phosphate-buffered saline (PBS) before sampling. On the first sampling day, sampling was conducted over a 30-min period. On the second sampling day, sampling was conducted for 60 min in order to increase yield. For the longer sampling period, the impinger solution was replenished with distilled deionized H_2_O to maintain the sampler collection efficiency (Lin et al. 1997) and avoid increasing liquid salinity. All sampling equipment was placed on top of a table (1.5 m from the ground) within an empty swine stall located within the facility approximately 30 m from the south wall of the swine facility where air exits through ventilation fans. At the time of sampling, four of eight 32-inch ventilation fans were in operation to maintain a farm-operator–designated target temperature of 21°C within the facility. Temperature and relative humidity were monitored throughout the sampling periods and were 22°C ± 1°C and 76 ± 4% respectively. Impingers were stored and transported back to the laboratory at 4°C.

### Bacterial isolation and speciation.

Approximately 3 hr after the last air sample was collected, impinger liquid samples were analyzed in the laboratory. Because no standard method exists regarding the isolation of *Enterococcus* from air, the standard methods used for the isolation of *Enterococcus* from recreational water were modified to accommodate the air samples [U.S. Environmental Protection Agency (EPA) 2000]. All broths and agars were obtained from Becton Dickinson (Sparks, MD). Three 10-fold dilutions (using PBS as the diluent) of the impinger samples were plated (100 μL/plate) in duplicate on mE agar. Negative control plates were made by plating 100 μL of both the replenishing fluid that was transported to the site and the dilution liquid. All plates were incubated for 48 hr at 41.5°C under aerobic conditions. All resulting colonies were counted, and counts from dilution plates containing 30–300 CFU were used in back-calculations to determine the concentration of isolated bacteria per cubic meter of air within the swine CAFO. Colonies from sample dilution plates that ranged from pink to red in color (indicative of *Enterococcus* colonies) were streaked onto Enterococcosel agar and incubated for 24 hr at 41.5°C under aerobic conditions. CFUs characteristic of *Enterococcus* that formed a black precipitate on the Enterococcosel agar plates were considered presumptive *Enterococcus* (U.S. EPA 2000). Presumptive *Enterococcus* isolates were archived in a 20% glycerol, tryptic soy broth solution at –80°C for subsequent speciation and antimicrobial susceptibility testing.

All presumptive *Enterococcus* isolates, as well as the quality control strains *E. faecium* 19434 and *Enterococcus faecalis* 29212 (American Type Culture Collection, Manassas, VA), were streaked from –80°C archived stocks onto both tryptic soy agar and tryptic soy agar No. 2 with 5% defibrinated sheep blood (Quad Five, Ryegate, MT) and incubated for 24 hr at 37°C. All of the media formulations and test interpretations used have been described previously (Murray et al. 2003). Gram stains were prepared on all isolates to verify the presence of gram-positive cocci. Each isolate was tested for the production of catalase in the presence of 3% hydrogen peroxide. Catalase-positive isolates were identified as *Staphylococcus* species (except for one isolate, which was further tested for oxidase activity and identified as *Micrococcus luteus*). Each *Staphylococcus* isolate was inoculated onto 0.5 mL rabbit plasma (Becton Dickinson) to test for the production of coagulase. Catalase-negative isolates were differentiated further by pyrrolidonyl-arylamidase activity using Remel’s PYR kit (Remel, Lenexa, KS). The following biochemical tests were performed on the isolates displaying pyrrolidonyl-arylamidase activity: mannitol, arabinose, sorbitol, raffinose, lactose, and sucrose carbohydrate fermentation tests; arginine deamination; acidification of methyl-α-d-glucopyranoside; pyruvate utilization; and isolate pigmentation.

### Antimicrobial susceptibility testing.

Antimicrobial susceptibility testing was conducted using the minimal inhibitory concentration (MIC) agar dilution method [National Committee for Clinical Laboratory Standards (NCCLS) 2002]. *E. faecalis* 29212 was used as the quality control reference strain. Susceptibility to erythromycin, clindamycin, virginiamycin (streptogramin A and B combination), tetracycline, and vancomycin was tested. Erythromycin, clindamycin, tetracycline, and vancomycin were obtained from Sigma (St. Louis, MO). Virginiamycin was obtained from Research Products International Corp. (Mt. Prospect, IL). Concentrations of antibiotics tested ranged from 0.5 μg/mL to 256 μg/mL for erythromycin and tetracycline, 0.03 μg/mL to 128 μg/mL for clindamycin, 0.03 μg/mL to 32 μg/mL for virginiamycin, and 0.03 μg/mL to 64 μg/mL for vancomycin.

In preparation for the agar dilution tests, the air sample isolates, as well as the MIC reference strain *E. faecalis* 29212, were streaked from –80°C archived stocks onto tryptic soy agar No. 2 with 5% defibrinated sheep blood (QuadFive, Ryegate, MT) and incubated for 24 hr at 37°C. After 24 hr, each isolate was suspended in 3 mL Mueller-Hinton broth with a sterile cotton swab and adjusted to a 0.5 McFarland standard using a Vitek colorimeter (Hach, Loveland, CO). Two hundred microliters of each suspension was transferred to a well within a Cathra replicator plate (Oxoid Inc., Ogdensburg, NY) and replicated with 1-mm pins in accordance with NCCLS guidelines onto Mueller-Hinton agar plates that were previously prepared with the appropriate concentrations of antibiotics (NCCLS 2002). Plates were incubated for 24 hr at 37°C under aerobic conditions. After 24 hr, the plates were read manually and MICs were determined. Specifically, the MIC was recorded as the minimum antibiotic concentration that completely inhibited bacterial growth. According to the MIC, isolates were categorized as susceptible, intermediate, or resistant to each antibiotic using the following MIC breakpoints established by the NCCLS for *Enterococcus*: erythromycin, susceptible ≤ 0.5 μg/mL, intermediate 1–4 μg/mL, and resistant ≥ 8 μg/mL; clindamycin, susceptible ≤ 0.5 μg/mL, intermediate 1–2 μg/mL, and resistant ≥ 4 μg/mL; virginiamycin, susceptible ≤ 1 μg/mL, intermediate 2 μg/mL, and resistant ≥ 4 μg/mL; tetracycline, susceptible ≤ 4 μg/mL, intermediate 8 μg/mL, and resistant ≥ 16 μg/mL; and vancomycin, susceptible ≤ 4 μg/mL, intermediate 8–16 μg/mL, and resistant ≥ 32 μg/mL (NCCLS 2002).

## Results

### Bacterial concentrations in air and bacterial identification.

The mean concentration of presumptive *Enterococcus* present in the air of the swine CAFO on both 9 December 2003 and 5 January 2004 was 4 × 10^4^ CFU/m^3^. After bacterial speciation was completed on 137 presumptive *Enterococcus* isolates, only 47 out of 137 isolates (34%) were confirmed to be *Enterococcus* (Table 1). Forty-four isolates (32%) were identified as staphylococci, 45 isolates (33%) were viridans group streptococci, and 1 isolate was identified as *Micrococcus luteus* (Table 1).

### Antibiotic resistance.

Ninety-eight percent (121 of 124) of the bacterial isolates that grew successfully during the antimicrobial susceptibility tests were resistant to high levels of at least two antibiotics commonly used in swine production (erythromycin, clindamycin, virginiamycin, or tetracycline), and 93% of the isolates (115 of 124) were resistant to three antibiotics commonly used in swine production. Individually, 98% of the isolates were resistant to erythromycin, 94% were resistant to clindamycin, 90% were resistant to tetracycline, and 37% were resistant to virginiamycin. None of the isolates displayed resistance to vancomycin. Because none of the *E. avium*, *E. pseudoavium*, or *E. raffinosus* isolates (all belonging to the *Enterococcus* physiologic group I) grew successfully on the control or antibiotic-amended MIC plates after being suspended as 0.5 McFarland standard solutions, MIC data for these isolates were not determined. MIC distributions among all other isolates were similar for erythromycin, clindamycin, tetracycline, and vancomycin, regardless of bacterial genus or species (Tables 2 and 3). For instance, across all organisms, most isolates (96%) had MICs > 256 μg/mL for erythromycin (Tables 2 and 3). In contrast, resistance to virginiamycin was more prevalent among coagulase-negative staphylococci versus *Enterococcus* or *Streptococcus* isolates (Tables 2 and 3). Phenotypes of antibiotic resistance among the bacterial isolates appear in Table 4.

## Discussion

In this study, multidrug-resistant *Enterococcus*, coagulase-negative staphylcocci, and viridans group streptococci were isolated from the air of a swine CAFO. Ninety-eight percent of the isolates were resistant to at least two of the following antibiotics: erythromycin, clindamycin, virginiamycin, and tetracycline, all of which are approved for use in swine production for growth promotion. In contrast, none of the isolates were resistant to vancomycin, which has never been approved for use in swine production in the United States. These results support the findings of previous reports that nontherapeutic use of antibiotics results in the presence of antibiotic-resistant bacteria in swine (Aarestrup et al. 2000a, 2000b; Bager et al. 1997; Jensen et al. 2002; Wegener et al. 1999). In addition, these results provide evidence that in the absence of nontherapeutic antibiotic use—vancomycin in this case—no resistance is detected among bacteria present in the swine environment.

Furthermore, these findings suggest that, in addition to the ingestion of retail pork products (Gambarotto et al. 2001; Hayes et al. 2003; Sorensen et al. 2001; White et al. 2001) and surface and groundwaters in the vicinity of swine CAFOs (Campagnolo et al. 2002; Chee-Sanford et al. 2001), the inhalation of air within swine operations may serve as another exposure pathway for the transfer of multidrug-resistant bacteria from swine to humans. These data are especially relevant to the health of swine CAFO workers, their direct contacts in the community, and possibly nearby neighbors of swine CAFOs.

The types of bacteria detected within the air of the swine facility investigated in this study are associated with a variety of human infections. *Enterococcus*, particularly some of the species isolated in this study including *E. faecalis* and *E. faecium*, has emerged as one of the leading causes of nosocomial bacteremias, urinary tract infections, and wound infections in the United States (NNIS 2001). Similarly, coagulase-negative staphylococci are the third most common causes of nosocomial infections and the most common causes of nosocomial bacteremias. The presence of multidrug-resistant *Enterococcus* and coagulase-negative staphylococci in patients significantly limits the treatment options available for these life-threatening infections. Although viridans group streptococci are part of the normal flora of the human respiratory tract, they also have been implicated as the cause of infective endocarditis and life-threatening septicemias in neutropenic patients. In addition, viridans group streptococci have been implicated as reservoirs of erythromycin-resistance genes, possibly capable of transferring resistance determinants to more pathogenic species including *Streptococcus pneumoniae* and *Streptococcus pyogenes* (Bryskier 2002).

Of particular concern to the health of individuals with direct or indirect contact with swine environments is the finding of virginiamycin-resistant gram-positive bacteria in the air of the swine CAFO. Virginiamycin, a streptogramin A and B combination, which has been used extensively as a growth promoter in swine, is an analog to quinupristin-dalfopristin, an injectable streptogramin A and B combination that is often the drug of last resort for multidrug-resistant gram-positive infections characterized by methicillin-resistant *Staphylococcus aureus* and glycopeptide-resistant *E. faecium* and coagulase-negative staphylococci (Johnson and Livermore 1999). Bacteria expressing resistance to virginiamycin are cross-resistant to quinupristin-dalfopristin, and a previous study has suggested that the transfer of streptogramin-resistant *Enterococcus* can occur between animals and humans in the livestock environment (Jensen et al. 1998). Thus, the inhalation of virginiamycin-resistant gram-positive bacteria in the swine environment could contribute to the appearance of quinupristin-dalfopristin–resistant gram-positive infections in humans, leaving few or no treatment options for the affected individual.

The finding of airborne clindamycin-resistant gram-positive bacteria in this study also is a potential concern to public health. Clindamycin is indicated for the treatment of human staphylococcal and streptococcal pneumonia (among other aerobic and anaerobic infections). Specifically, clindamycin has been used for the treatment of community-acquired methicillin-resistant *S. aureus* (Marcinak and Frank 2003). Clindamycin also has been shown to be significantly more potent than penicillin in inhibiting both invasive and noninvasive group A streptococci such as *S. pyogenes* (Mascini et al. 2001). The findings of airborne clindamycin-resistant coagulase-negative staphylococci and viridans group streptococci in the swine environment raise the question as to whether these organisms could serve as reservoirs of clindamycin-resistant genes [as well as reservoirs of erythromycin-resistant genes (Bryskier 2002)], passing on clindamycin resistance determinants to more pathogenic species as described above.

Furthermore, exposure to virginiamycin-, erythromycin-, clindamycin-, and tetracycline-resistant *Enterococcus*, coagulase-negative staphylococci, and viridans group streptococci through the inhalation of contaminated air could lead to the colonization of these multidrug-resistant organisms in both the nasal passages (Aubry-Damon 2004) and the lungs of swine CAFO workers, potentially making the workers themselves reservoirs of antibiotic-resistant organisms. Coexposures to other aerosols and gases in the swine environment such as organic dusts, molds, and ammonia have been shown to induce symptoms associated with chronic bronchitis, including a persistent cough characterized by expectoration (Mackiewicz 1998). The presence of this type of cough can increase the potential for secondary spread of antibiotic-resistant organisms into the community, where additional individuals could serve as reservoirs of multidrug-resistant bacteria.

Moreover, the tunnel-ventilated design of swine CAFOs, which moves air outside of the facilities at a high flow rate, could create a situation where neighbors living downwind of the ventilation fans also could be directly exposed to airborne multidrug-resistant bacteria. An epidemiologic study by Wing and Wolf (2000) indicated that people who live in the vicinity of swine CAFOs experience elevated rates of headaches, runny noses, sore throats, excessive coughing, and diarrhea compared with people living in communities that are not situated near livestock operations. The findings of airborne multidrug-resistant bacteria in a swine CAFO in our study raise the question as to whether airborne bacteria also could travel beyond the confines of the swine CAFO on ventilation fan air currents, directly contacting nearby neighbors and potentially contributing to health effects such as those observed in the Wing and Wolf study. Because populations living in areas where swine CAFOs are built already may experience higher rates of certain diseases because of lack of access to appropriate health care (Weber et al. 1989), investigating airborne exposures to multidrug-resistant bacteria among these at-risk populations is an important area for future research.

In addition to potential airborne exposures occurring among individuals living near swine CAFOs, the results of this study could have broader public health implications. Specifically, one may question whether airborne exposures to multidrug-resistant bacteria could be occurring and contributing to health problems around other environmental sources of animal or human waste, including land application areas for animal waste and human sludge, and human wastewater treatment facilities. Endotoxins, exotoxins, and other chemical components in dusts associated with animal waste and human sludge have been linked to hypersensitivity reactions among individuals living near land application areas (Lewis and Gattie 2002). These reactions have been shown to result in increased susceptibility to serious respiratory infections, including those caused by *S. aureus* (Lewis and Gattie 2002). Thus, the presence of high concentrations of multidrug-resistant staphylococci and other bacterial pathogens amidst endotoxin-containing dust from animal and human waste could pose unique health concerns to people living near land application areas.

## Conclusions

In summation, the findings of this study suggest that the inhalation of air from swine CAFOs may serve as an additional environmental exposure pathway for the transfer of multidrug-resistant bacterial pathogens from swine to humans. Given the growing interest in reservoirs of antibiotic resistance genes associated with large-scale livestock operations (Nandi et al. 2004), our findings in this investigation emphasize the importance of studying multiple genera of bacteria in different environmental media as sources of human exposure to antibiotic resistance genes.

## Figures and Tables

**Table 1:** Airborne bacteria isolated from a swine CAFO using methods for the isolation of *Enterococcus* species.

Bacteria	No. of isolates (%)
*Enterococcus*	47 (34)
*E. avium*	5 (4)
*E. dispar*	4 (3)
*E. durans*	2 (1)
*E. faecalis*	6 (4)
*E. faecium*	1 (< 1)
*E. hirae*	14 (10)
*E. mundtii*	1 (< 1)
*E. pseudoavium*	2 (1)
*E. raffinosus*	1 (< 1)
Other	11 (8)
*Staphylococcus*	44 (32)
*S. aureus*	1 (< 1)
Coagulase-negative staphylococci	43 (31)
*Streptococcus*	
Viridans group streptococci	45 (33)
*Micrococcus luteus*	1 (< 1)
Total	137 (100)

**Table 2:** MIC distributions for five antibiotics observed in airborne *Enterococcus* collected from a swine CAFO.

	No. of bacterial isolates with the following MICs (μg/mL)													
Bacteria, antibiotic	≤ 0.5	1	2	4	8	16	32	64	128	256	> 256	%S	%I	%R
*Enterococcus* (*n* = 38)														
Erythromycin			1								37	0	3	97
Clindamycin	1				1	1	1	3	8	23*^a^*		3	0	97
Virginiamycin	19	5	5	9								63	13	24
Tetracycline				1	2	7	6	17	5			3	5	92
Vancomycin	38											100	0	0
*E. dispar* (*n* = 4)															
Erythromycin											4	0	0	100
Clindamycin								1	1	2*^a^*		0	0	100
Virginiamycin	4											100	0	0
Tetracycline								3	1			0	0	100
Vancomycin	4											100	0	0
*E. durans* (*n* = 2)															
Erythromycin											2	0	0	100
Clindamycin									1	1*^a^*		0	0	100
Virginiamycin			1	1								0	50	50
Tetracycline				1	1							50	50	0
Vancomycin	2											100	0	0
*E. faecalis* (*n* = 6)															
Erythromycin	1										5	17	0	83
Clindamycin	1				1				2	2*^a^*		17	0	83
Virginiamycin	2	3		1								83	0	17
Tetracycline						2	1	2	1			0	0	100
Vancomycin	6											100	0	0
*E. faecium* (*n* = 1)															
Erythromycin											1	0	0	100
Clindamycin									1			0	0	100
Virginiamycin				1								0	0	100
Tetracycline						1						0	0	100
Vancomycin	1											100	0	0
*E. hirae* (*n* = 14)															
Erythromycin											14	0	0	100
Clindamycin									2	12*^a^*		0	0	100
Virginiamycin	8		2	4								57	14	29
Tetracycline					1	2	1	8	2			0	7	93
Vancomycin	14											100	0	0
Other (*n* = 11)															
Erythromycin											11	0	0	100
Clindamycin						1	1	2	1	6*^a^*		0	0	100
Virginiamycin	5	2	2	2								64	18	18
Tetracycline						2	4	4	1			0	0	100
Vancomycin	11											100	0	0

Abbreviations: %I, percent intermediate; %R, percent resistant; %S, percent susceptible.

a MIC is > 128 μg/mL.

**Table 3:** MIC distributions for five antibiotics observed in airborne *Staphylococcus* and *Streptococcus* collected from a swine CAFO.

	Number of bacterial isolates with the following MICs (μg/mL)														
Bacteria, antibiotic	≤ 0.5	1	2	4	8	16	32	64	128	256	> 256	%S	%I	%R
*Staphylococcus* (*n* = 43)*^a^*														
*S. aureus* (*n* = 1)														
Erythromycin											1	0	0	100
Clindamycin										1*^b^*		0	0	100
Virginiamycin			1									0	100	0
Tetracycline								1				0	0	100
Vancomycin	1											100	0	0
Coagulase-negative														
staphylococci (*n* = 42)														
Erythromycin											42	0	0	100
Clindamycin		1							2	39*^b^*		0	2	98
Virginiamycin	4	2	2	21	13							14	5	81
Tetracycline		2		1		1	5	12	13	7	1	7	0	93
Vancomycin	7	3	30	2								100	0	0
*Streptococcus* (*n* = 43)*^c^*														
Viridans group streptococci														
Erythromycin			1			1	1				40	0	0	100
Clindamycin	2						2	2	9		28	5	0	95
Virginiamycin	29	7	4	3								84	9	7
Tetracycline	1					8	17	10	7			2	0	98
Vancomycin	43											100	0	0

Abbreviations: %I, percent intermediate; %R, percent resistant; %S, percent susceptible.

a Analyzed using the breakpoints for *Enterococcus*.

b MIC is > 128 μg/mL.

c Analyzed using the following breakpoints: erythromycin, susceptible ≤ 0.25 μg/mL, intermediate 0.5 μg/mL, and resistant ≥ 1.0 μg/mL; clindamycin, susceptible ≤ 0.5 μg/mL, intermediate 1–2 μg/mL, and resistant ≥ 4 μg/mL; virginiamycin, susceptible ≤ 1 μg/mL, intermediate 2 μg/mL, and resistant ≥ 4 μg/mL; tetracycline, susceptible ≤ 2 μg/mL, intermediate 4 μg/mL, and resistant ≥ 8 μg/mL; vancomycin, susceptible ≤ 1 μg/mL, intermediate and resistant not available (NCCLS 2002).

**Table 4:** Phenotypes of antibiotic resistance among airborne bacteria collected from a swine CAFO.

Bacteria	Antibiotic resistance pattern	No. of isolates (%)
*Enterococcus*		
*E. dispar* (*n* = 4)	Ery, Clin, Tet	4 (100)
*E. durans* (*n* = 2)	Ery, Clin	1 (50)
	Ery, Clin, Virg	1 (50)
*E. faecalis* (*n* = 6)	Tet	1 (17)
	Ery, Clin, Tet	4 (66)
	Ery, Clin, Tet, Virg	1 (17)
*E. faecium* (*n* = 1)	Ery, Clin, Tet, Virg	1 (100)
*E. hirae* (*n* = 14)	Ery, Clin	1 (7)
	Ery, Clin, Tet	9 (64)
	Ery, Clin, Tet, Virg	4 (29)
Other *Enterococcus* (*n* = 11)	Ery, Clin, Tet	9 (82)
	Ery, Clin, Tet, Virg	2 (18)
*Staphylococcus aureus* (*n* = 1)	Ery, Clin, Tet	1 (100)
Coagulase-negative staphylococci (*n* = 42)	Ery, Tet	1 (2)
	Ery, Clin, Tet	8 (19)
	Ery, Clin, Virg	6 (14)
	Ery, Virg, Tet	1 (2)
	Ery, Clin, Tet, Virg	26 (62)
Viridans group streptococci (*n* = 43)	Tet	2 (5)
	Ery, Clin	1 (2)
	Ery, Tet	2 (5)
	Ery, Clin, Tet	35 (81)
	Ery, Clin, Tet, Virg	3 (7)

Abbreviations: Clin, clindamycin; Ery, erythromycin; Tet, tetracycline; Virg, virginiamycin.

## References

[b1-ehp0113-000137] Aarestrup FM, Agerso Y, Gerner-Smidt P, Madsen M, Jensen LB (2000a). Comparison of antimicrobial resistance phenotypes and resistance genes in *Enterococcus faecalis* and *Enterococcus faecium* from humans in the community, broilers, and pigs in Denmark. Diagn Microbiol Infect Dis.

[b2-ehp0113-000137] Aarestrup FM, Kruse H, Tast E, Hammerum AM, Jensen LB (2000b). Associations between the use of antimicrobial agents for growth promotion and the occurrence of resistance among *Enterococcus faecium* from broilers and pigs in Denmark, Finland, and Norway. Microb Drug Resist.

[b3-ehp0113-000137] Aubry-Damon H, Grenet K, Sall-Ndiaye P, Che D, Cordeiro E, Bougnoux ME (2004). Antimicrobial resistance in commensal flora of pig farmers. Emerg Infect Dis.

[b4-ehp0113-000137] Bager F, Madsen M, Christensen J, Aarestrup FM (1997). Avoparcin used as a growth promoter is associated with the occurrence of vancomycin-resistant *Enterococcus faecium* on Danish poultry and pig farms. Prev Vet Med.

[b5-ehp0113-000137] Bryskier A (2002). Viridans group streptococci: a reservoir of resistant bacteria in oral cavities. Clin Microbiol Infect.

[b6-ehp0113-000137] Campagnolo ER, Johnson KR, Karpati A, Rubin CS, Kolpin DW, Meyer MT (2002). Antimicrobial residues in animal waste and water resources proximal to large-scale swine and poultry feeding operations. Sci Total Environ.

[b7-ehp0113-000137] Chee-Sanford JC, Aminov RI, Krapac IJ, Garrigues-Jeanjean N, Mackie RI (2001). Occurrence and diversity of tetracycline resistance genes in lagoons and groundwater underlying two swine production facilities. Appl Environ Microbiol.

[b8-ehp0113-000137] Clark S, Rylander R, Larsson L (1983). Airborne bacteria, endotoxin and fungi in dust in poultry and swine confinement buildings. Am Ind Hyg Assoc J.

[b9-ehp0113-000137] Cohen ML, Tauxe RV (1986). Drug-resistant *Salmonella* in the United States: an epidemiologic perspective. Science.

[b10-ehp0113-000137] Cormier Y, Tremblay G, Meriaux A, Brochu G, Lavoie J (1990). Airborne microbial contents in two types of swine confinement buildings in Quebec. Am Ind Hyg Assoc J.

[b11-ehp0113-000137] Crook B, Robertson JF, Glass SA, Botheroyd EM, Lacey J, Topping MD (1991). Airborne dust, ammonia, microorganisms, and antigens in pig confinement houses and the respiratory health of exposed farm workers. Am Ind Hyg Assoc J.

[b12-ehp0113-000137] Donabedian SM, Thal LA, Hershberger E, Perri MB, Chow JW, Bartlett P (2003). Molecular characterization of gentamicin-resistant *Enterococci* in the United States: evidence of spread from animals to humans through food. J Clin Microbiol.

[b13-ehp0113-000137] Environmental Defense 1997. Animal Waste Summary. New York:Environmental Defense. Available: http://www.hogwatch.org/maps/index [accessed 15 June 2004].

[b14-ehp0113-000137] Food and Drug Administration 2004. FDA Approved Animal Drug Products. Blacksburg, VA:Drug Information Laboratory, Virginia/Maryland Regional College of Veterinary Medicine.

[b15-ehp0113-000137] Gambarotto K, Ploy MC, Dupron F, Giangiobbe M, Denis F (2001). Occurrence of vancomycin-resistant enterococci in pork and poultry products from a cattle-rearing area of France. J Clin Microbiol.

[b16-ehp0113-000137] Gorbach SL (2001). Antimicrobial use in animal feed—time to stop. N Engl J Med.

[b17-ehp0113-000137] Hamscher G, Pawelzick HT, Sczesny S, Nau H, Hartung J (2003). Antibiotics in dust originating from a pig-fattening farm: a new source of health hazard for farmers?. Environ Health Perspect.

[b18-ehp0113-000137] Hayes JR, English LL, Carter PJ, Proescholdt T, Lee KY, Wagner DD (2003). Prevalence and antimicrobial resistance of enterococcus species isolated from retail meats. Appl Environ Microbiol.

[b19-ehp0113-000137] Institute of Medicine 1998. Antimicrobial Resistance: Issues and Options, Workshop Report, Forum on Emerging Infections. Washington, DC:National Academy Press. 23035315

[b20-ehp0113-000137] Jensen LB, Hammerum AM, Aarestrup FM, van den Bogaard AE, Stobberingh EE (1998). Occurrence of *satA* and *vgb* genes in streptogramin-resistant *Enterococcus faecium* isolates of animal and human origins in the Netherlands. Antimicrob Agents Chemother.

[b21-ehp0113-000137] Jensen LB, Hammerum AM, Bager F, Aarestrup FM (2002). Streptogramin resistance among *Enterococcus faecium* isolated from production animals in Denmark in 1997. Microb Drug Resist.

[b22-ehp0113-000137] Johnson AP, Livermore DM (1999). Quinupristin/dalfopristin, a new addition to the antimicrobial arsenal. Lancet.

[b23-ehp0113-000137] Lewis DL, Gattie DK (2002). Pathogen risks from applying sewage sludge to land. Environ Sci Technol.

[b24-ehp0113-000137] Lin X, Willeke K, Ulevicius V, Grinshpun S (1997). Effect of sampling time on the collection efficiency of all-glass impingers. Am Ind Hyg Assoc J.

[b25-ehp0113-000137] Mackiewicz B (1998). Study on exposure of pig farm workers to bioaerosols, immunologic reactivity and health effects. Ann Agric Environ Med.

[b26-ehp0113-000137] Marcinak JF, Frank AL (2003). Treatment of community-acquired methicillin-resistant Staphylococcus aureus in children. Curr Opin Infect Dis.

[b27-ehp0113-000137] Mascini EM, Janszea M, Schoulsb LM, Verhoefa J, Van Dijk H (2001). Penicillin and clindamycin differentially inhibit the production of pyrogenic exotoxins A and B by group A streptococci. Int J Antimicrob Agents.

[b28-ehp0113-000137] Mellon M Benbrook C Benbrook KL 2001. Hogging It: Estimates of Antimicrobial Abuse in Livestock. Cambridge, MA:Union of Concerned Scientists Publications.

[b29-ehp0113-000137] Murray PR Baron EJ Jorgensen JH Pfaller MA Yolken RH 2003. Manual of Clinical Microbiology. 8th ed. Washington, DC:American Society for Microbiology Press.

[b30-ehp0113-000137] Nandi S, Maurer JJ, Hofacre C, Summers AO (2004). Gram-positive bacteria are a major reservoir of class 1 antibiotic resistance integrons in poultry litter. Proc Natl Acad Sci USA.

[b31-ehp0113-000137] National Research Council 1999. The Use of Drugs in Food Animals: Benefits and Risks. Washington, DC:National Academy Press. 25121246

[b32-ehp0113-000137] NCCLS 2002. Performance Standards for Antimicrobial Disk and Dilution Susceptibility Tests for Bacteria Isolates from Animals; Approved Standard. 2nd ed. M31-A2. Wayne, PA:National Committee for Clinical Laboratory Standards.

[b33-ehp0113-000137] NNIS (2001). National Nosocomial Infections Surveillance (NNIS) system report, data summary from January 1992–June 2001. Am J Infect Control.

[b34-ehp0113-000137] Predicala BZ, Urban JE, Maghirang RG, Jerez SB, Goodband RD (2002). Assessment of bioaerosols in swine barns by filtration and impaction. Curr Microbiol.

[b35-ehp0113-000137] Sorensen TL, Blom M, Monnet DL, Frimodt-Moller N, Poulsen RL, Espersen F (2001). Transient intestinal carriage after ingestion of antibiotic-resistant *Enterococcus faecium* from chicken and pork. N Engl J Med.

[b36-ehp0113-000137] U.S. EPA 2000. Improved Enumeration Methods for the Recreational Water Quality Indicators: Enterococci, and *Escherichia coli* EPA/821/R-97/004. Washington, DC:U.S. Environmental Protection Agency.

[b37-ehp0113-000137] van den Boogard AE, Stobberingh EE (1999). Antibiotic usage in animals: impact on bacterial resistance and public health. Drugs.

[b38-ehp0113-000137] Weber D, Rutala W, Samsa G, Sarubbi F, King L (1989). Epidemiology of tuberculosis in North Carolina, 1966 to 1986: analysis of demographic features, geographic variation, AIDS, migrant workers, and site of infection. South Med J.

[b39-ehp0113-000137] Wegener HC (2003). Antibiotics in animal feed and their role in resistance development. Curr Opin Microbiol.

[b40-ehp0113-000137] Wegener HC, Aarestrup FM, Jensen LB, Hammerum AM, Bager F (1999). Use of antimicrobial growth promoters in food animals and *Enterococcus faecium* resistance to therapeutic antimicrobial drugs in Europe. Emerg Infect Dis.

[b41-ehp0113-000137] White DG, Zhao S, Sudler R, Ayers S, Friedman S, Chen S (2001). The isolation of antibiotic-resistant salmonella from retail ground meats. N Engl J Med.

[b42-ehp0113-000137] Wing S, Wolf S (2000). Intensive livestock operations, health, and quality of life among eastern North Carolina residents. Environ Health Perspect.

[b43-ehp0113-000137] Zahn JA, Anhalt J, Boyd E (2001). Evidence for transfer of tylosin and tylosin-resistant bacteria in air from swine production facilities using sub-therapeutic concentrations of tylan in feed [Abstract]. J Anim Sci.

